# Supplementary material for: Identification of photoperiod-induced specific miRNAs in the adrenal glands of Sunite sheep (Ovis aries)
Source: Front Vet Sci. 2022 Jul 22;9:888207. doi: 10.3389/fvets.2022.888207 (PMC9354845; doi:10.3389/fvets.2022.888207)
Supplement: Supplementary file 1 [file Data_Sheet_1.ZIP › Supplementary file/miRDeep2 file.zip!/miRDeep2 file/Know_Structure/SP42A2/oar-mir-26b.pdf]

miRBase precursor : oar-mir-26b
Total read count : 202362
oar-miR-26b read count : 202165
remaining reads : 197

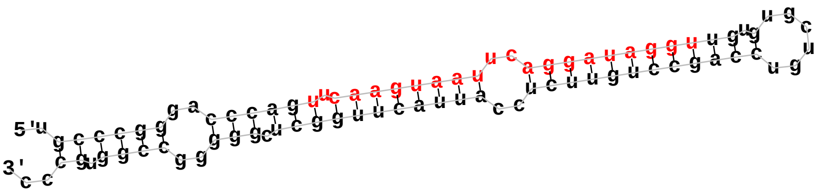

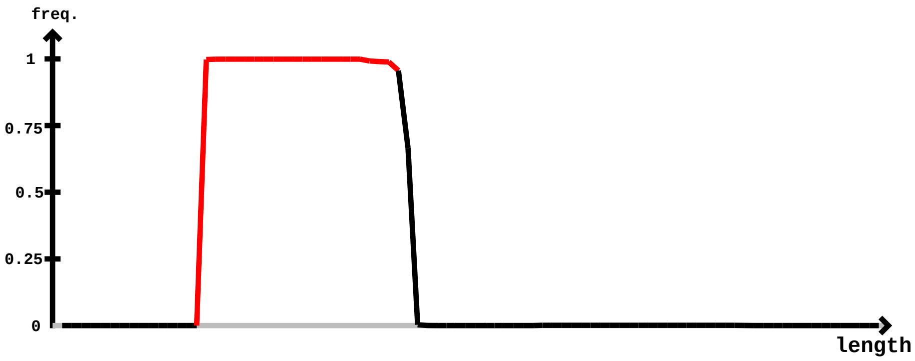


```
                  oar-miR-26b
5'- ugcccgggacccaguucaaguaauucaggauagguugugugcuguccagccuguucuccauuacuuggcucgggggccggugccc  -3'    exp
    .((((((..((((((.((((((((..(((((((((((.(......)))))))))))).)))))))))))).))).)))).))..    reads    mm    sample
    .............guucaaguaauucaggauagg...................................................    1        0     seq
    .............guucaaguaauucaggauaggu..................................................    2        0     seq
    .............guucaaguaauucaggauagguu.................................................    1        0     seq
    ..............uucaaguaauucaggau......................................................    1095     0     seq
    ..............uCcaaguaauucaggau......................................................    4        1     seq
    ..............uucaaguaauucaggaA......................................................    28       1     seq
    ..............uucaaguaauucaggUu......................................................    3        1     seq
    ..............uucaaguGauucaggau......................................................    2        1     seq
    ..............uuUaaguaauucaggau......................................................    2        1     seq
    ..............uucCaguaauucaggau......................................................    83       1     seq
    ..............uucaaguaauuUaggau......................................................    1        1     seq
    ..............uucaaguaCuucaggau......................................................    1        1     seq
    ..............uucaaguaauucaggaC......................................................    140      1     seq
    ..............uucaGguaauucaggau......................................................    7        1     seq
    ..............uucaagCaauucaggau......................................................    2        1     seq
    ..............uucaaguaauucagUau......................................................    1        1     seq
    ..............uucaaguaauucaggCu......................................................    1        1     seq
    ..............uucaaguaaCucaggau......................................................    5        1     seq
    ..............uucaaguaauucagAau......................................................    3        1     seq
    ..............Aucaaguaauucaggau......................................................    7        1     seq
    ..............uucaaguaauucaggaG......................................................    12       1     seq
    ..............uucaUguaauucaggau......................................................    1        1     seq
    ..............uucCaguaauucaggaua.....................................................    36       1     seq
    ..............uucaaguaauucaggaCa.....................................................    5        1     seq
    ..............uucaaguaauucaggaua.....................................................    410      0     seq
    ..............uucaaguaauucaggaAa.....................................................    5        1     seq
    ..............uucaaguaauuUaggaua.....................................................    2        1     seq
    ..............uucaaguaauucaggauag....................................................    283      0     seq
    ..............uucaaguaauucaggauagg...................................................    6547     0     seq
    ..............uucaaguaauucaggauaggu..................................................    58737    0     seq
    ..............uucaaguaauucaggauagguu.................................................    133850   0     seq
    ..............uucaaguaauucaggauagguug................................................    547      0     seq
    ..............uucaaguaauucaggauagguugu...............................................    62       0     seq
    ...............ucaaguaauucaggaua.....................................................    2        0     seq
    ...............ucaaguaauucaggauagg...................................................    6        0     seq
```

oar-miR-26b

```
ugcccgggacccaguucaaguaauucaggauagguugugugcuguccagccuguucuccauuacuuggcucgggggccggugccc
...............ucaaguaauucaggauaggu..................................................  54    0    seq
...............ucaaguaauucaggauagguu.................................................  158   0    seq
...............ucaaguaauucaggauagguug................................................  8     0    seq
................caaguaauucaggauaggu..................................................  8     0    seq
................caaguaauucaggauagguu.................................................  28    0    seq
................caaguaauucaggauagguugu...............................................  1     0    seq
.................aaguaauucaggauagguu.................................................  14    0    seq
................................................gccuguucuccauuacuugg.................  1     0    seq
.................................................ccuguucuccauuacuugg.................  1     0    seq
.................................................ccuguucuccauuacuuggc................  6     0    seq
.................................................ccuguucuccauuacuuggcu...............  103   0    seq
.................................................ccuguucuccauuacuuggcuc..............  78    0    seq
.................................................ccuguucuccauuacuuggcucg.............  7     0    seq
..................................................cuguucuccauuacuuggcuc..............  1     0    seq
```
